# Prevalence and Predictors of Missed Nursing Care in Greek Public Hospitals: A National Cross‐Sectional Study

**DOI:** 10.1155/jonm/3078316

**Published:** 2026-04-30

**Authors:** Alexandra Giannarou, Michael Igoumenidis, Nikos Stefanopoulos, Anastasios Tzenalis, Petros Galanis, Stefania Chiappinotto, Alvisa Palese

**Affiliations:** ^1^ Department of Nursing, University of Patras, Patras, Greece, upatras.gr; ^2^ Department of Nursing, University of Athens, Athens, Greece, uoa.gr; ^3^ Department of Medicine, University of Udine, Udine, Italy, uniud.it

**Keywords:** Missed Nursing Care, nursing quality, predictors, standards for quality care, understaffing

## Abstract

**Aim:**

To describe the prevalence of Missed Nursing Care and its predictors in Greek public hospitals.

**Background:**

Missed Nursing Care is defined as any aspect of required patient care that is omitted or delayed. Despite the available studies, little is still known in countries with significant nursing shortages, such as Greece, where 2.23 registered and assistant nurses per 1000 population have been reported, significantly below the EU‐27 average.

**Methods:**

A national cross‐sectional study was conducted in 28 of 124 public hospitals in Greece. Nurses and nursing assistants working in medical or surgical units, providing direct care to adult patients, and with at least 3 months of experience were eligible. The MISSCARE Survey Part A (5‐point Likert scale; 1 = never, 5 = always missed), Part B (reasons, four‐point Likert scale; 1 = not significant, 4 = significant reason) and the Practice Environment Scale of the Nurse Work Index (4‐point Likert scale; 1 = totally agree, 4 = totally disagree) were used. Descriptive and inferential statistics were applied.

**Results:**

A total of 676 nurses participated. The Missed Nursing Care Part A total score was 2.06 (±0.65), with patients’ daily activities (mean = 2.32 ± 0.73) receiving higher scores than activities related to health status and treatments (mean = 1.80 ± 0.63). The overall score on the Practice Environment Scale of the Nurse Work Index was 2.53 (±0.49). Multiple linear regression analysis showed that issues in nursing care standards for quality of care, staffing adequacy and communication within the team were the most significant predictors of Missed Nursing Care.

**Conclusion:**

Missed Nursing Care is a major problem in Greek hospitals. Inadequate staffing is a key factor in missed care according to nurses’ perceptions. Increasing nursing staff, along with implementing standards for nursing care and improving communication among team members, will enhance the quality of health services in Greece.

**Implications for Nursing Management:**

Strengthening staffing levels and reinforcing nursing standards are essential strategies for reducing Missed Care in Greek public hospitals.

## 1. Introduction

Over the past 2 decades, global interest in the phenomenon of Missed Nursing Care (MNC) and its various dimensions has increased. Several studies confirm the correlation between MNC and adverse events in hospitalised patients, such as falls, pressure ulcers, infections and medication errors [1]. A recent systematic review and meta‐analysis reports a median global prevalence of 56.4%, with the most frequent reasons for MNC being ‘unexpected rise in patient volume and/or acuity on the unit’, ‘inadequate number of staff’ and ‘urgent patient situation’ [2]. However, the perspective of a single nation on this phenomenon, especially those with documented important shortages in nursing care such as Greece, is still missing, leading to a gap in the available literature.

## 2. Background

Studies have consistently shown that MNC is more likely to occur when nurses are overburdened. In these situations, nurses must prioritise some tasks over others, often omitting non‐urgent but essential care such as patient education, emotional support or hygiene care [3]. Insufficient staffing levels reduce nurses’ capacity to deliver timely and comprehensive care, especially in high‐acuity settings, leading to the prioritisation of certain tasks and resulting in omissions [4]. Aiken and colleagues documented that each additional patient per nurse increases the likelihood of care being missed due to time constraints [5]. Similarly, more recent studies across various healthcare systems have indicated that understaffed units report higher incidences of omitted interventions, such as patient ambulation, hygiene and discharge planning [6, 7]. Moreover, inadequate staffing not only compromises patient outcomes, such as increased rates of infections, falls and readmissions, but also increases nurse dissatisfaction, burnout and intention to leave the profession [3].

Within the Greek healthcare system, hospitals are often characterised by chronic nurse understaffing, limited resources and high bed‐to‐nurse ratios [8] with an overall ratio of 2.23 nurses per 1000 population in 2024, significantly below the EU27 average of 7.52 as documented recently [9]. Staff inadequacy, combined with high job demands and limited administrative support, creates a context in which missed care is likely to occur daily. Greek nurses also frequently report high levels of burnout, turnover intention, low job satisfaction and limited opportunities for professional development [10], all of which are known to correlate with care omissions [3]. The healthcare system has been under significant strain, especially following the financial crisis of the previous decade and the additional burdens imposed by the COVID‐19 pandemic [11].

Cultural and organisational factors may further complicate the situation. In the traditionally hierarchical structure of Greek hospitals, nurses often have limited decision‐making authority, including in prioritisation, and interprofessional collaboration is weak, hindering efficient communication and resource allocation [10]. Additionally, it is not uncommon to observe overlapping responsibilities between registered nurses (RNs) and Nursing Assistants (NAs). Officially, RNs and NAs differ in several ways. In terms of education, RNs hold a 4‐year university degree, whereas NAs complete a shorter, 2‐year vocational training programme. Regarding supervision, RNs work autonomously and often supervise NAs who do not have supervisory roles. In terms of the scope of practice, RNs perform a range of duties, such as medicine administration, care coordination and documentation, whereas NAs provide auxiliary and basic care, such as assistance with daily activities and measuring vital signs. However, even though they have different job responsibilities, due to ongoing nursing shortages, RNs and NAs may sometimes perform similar tasks in practice [12].

Overall, despite these challenges, there are very few studies [13, 14] focusing specifically on MNC within the Greek context, where cultural, organisational and economic factors may uniquely influence the delivery of nursing care. Investigating these predictors within the Greek healthcare system is therefore crucial for developing targeted interventions and policies to improve care quality. Additionally, as the global nursing shortage persists, investigating implications of staffing inadequacies across countries may clarify variations in MNC and inform the need for country‐specific policies.

## 3. Aim

The aim of this study was to describe MNC and its predictors among public hospital–based nurses in Greece. By examining variables such as nursing demographics and the quality of the work environment, the study seeks to provide evidence‐based insights into the root causes of MNC. Overall, the findings are expected to contribute to the international literature on MNC and to the efforts of Greek healthcare stakeholders to address structural and operational barriers to safe and effective nursing care.

## 4. Methods

### 4.1. Study Design

A cross‐sectional study was performed and here reported according to the guidelines for STrengthening the Reporting of OBservational studies in Epidemiology (STROBE) [15].

### 4.2. Setting and Participants

As this was a nationwide study, the aim was to include hospitals from as many different organisational healthcare regions as possible. In Greece, there are seven regional health authorities that coordinate, supervise and control the operations of all health service providers within their territories: Attica, Piraeus/Islands, Western Greece/Peloponnese, Central Greece, Western Macedonia, Eastern Macedonia and Thrace, and Crete. A list of public hospitals operating in each regional health authority was therefore compiled; private hospitals were not eligible. A random stratified sample of 30 public hospitals (out of a possible 124) was selected to ensure a representative mix of large and small hospitals (with 300 beds or more, and fewer than 300 beds, respectively) and region. Invitations were sent to all 30 hospitals, along with requests for permission to access the hospital facilities, which were granted by the ethical committees of each hospital. In the end, six out of the seven territories participated, as there were issues of proximity with the island of Crete, where the two identified hospitals declined to participate. Therefore, the final number of participating hospitals was 28.

All RNs and NAs employed in general medical and surgical wards of the selected hospitals, providing direct care to adult patients, with at least 3 months of work experience in the medical or surgical unit, either as permanent or temporary staff, were eligible. Both RNs and NAs were included to better understand the phenomenon of MNC in a realistic way due to the overlapping responsibilities previously explained. Nursing students, and RNs or NAs on maternity or sick leave, were excluded.

### 4.3. Data Collection Instruments

The Greek versions of the MISSCARE survey [16] and the Practice Environment Scale of the Nursing Work Index (PES‐NWI) [17] were used. The MISSCARE survey was validated by Papastavrou et al. [17], demonstrating satisfactory internal consistency reliability for both parts of the questionnaire: Part A (measuring missed interventions; Cronbach’s alpha = 0.957) and Part B (measuring underlying reasons; Cronbach’s alpha = 0.936). The PES‐NWI was previously validated by Prezerakos et al. [17]. Cronbach’s alpha for the instrument’s subscales ranged from 0.60 to 0.80, and for the overall PES‐NWI, it was 0.86, indicating acceptable reliability. Permission to use both instruments was obtained from Dr Papastavrou and Dr Moisoglou. For the purpose of our study, these instruments were supplemented with an ad hoc form to collect demographic and professional data (e.g., age, education) developed by the research team and piloted before its ample use.

#### 4.3.1. The MISSCARE Survey

The MISSCARE survey was developed by Kalisch and Williams in 2009 [4] and is recognised as the most widely used instrument in the field of MNC measurement, with good psychometric properties [18]. Its Part A consists of 24 questions and measures nurses’ perceptions of missed elements/interventions in the care they provide, asking them to indicate how often, during their last shift, they left any aspect of care missed. According to studies in the field [19], we divided the items into two dimensions: (1) MNC related to patients’ activities of daily living and (2) MNC related to patients’ current health status and treatment. The survey uses a 5‐point Likert scale (1 = never, 5 = always), and the total score may range from 24 to 120. In our study, the internal reliability, as measured by Cronbach’s alpha, was 0.933 for Part A: 0.888 for MNC related to patients’ activities of daily living and 0.887 for MNC related to patients’ current health status and treatment. Part B of the MISSCARE survey asks participants to report the reasons for missed care from a list of 17 questions using a 4‐point Likert scale according to the importance assigned to each reason (1, not significant reason; 4, significant reason). As indicated in the literature (Palese et al., 2021), reasons are categorised as related to ‘*Communication and Teamwork*’, ‘*Labour resources*’ and ‘*Material resources*’, and the total score may range from 17 to 68. In our study, internal reliability, as measured by Cronbach’s alpha, was 0.878 for Part B of the survey (0.846 for ‘*Communication and Teamwork*’, 0.768 for ‘*Labour resources*’ and 0.825 for ‘*Material resources*’).

#### 4.3.2. The Practice Environment Scale of the Nursing Work Index

The PES‐NWI is the most widely used instrument for assessing the nursing practice environment [20]. It comprises 32 items across five subscales reflecting the professional nursing work–life environment: (1) ‘*Nurse manager ability*’ (8 items), (2) ‘*Staffing and resource adequacy*’ (4 items), (3) ‘*Collegial nurse-physician relationships*’ (3 items), (4) ‘*Nursing foundations for quality of care*’ (8 items) and (5) ‘*Nurse participation in hospital affairs*’ (9 items). Each item is rated on a four‐point Likert scale (1—totally agree, 4—totally disagree). In our study, the internal reliability, as measured by Cronbach’s alpha, was 0.944 for the PES‐NWI overall (0.770 for ‘*Nurse manager ability*’, 0.713 for ‘*Staffing and resource adequacy*’, 0.898 for ‘*Collegial nurse-physician relationships*’, 0.803 for ‘*Nursing foundations for quality of care*’ and 0.850 for ‘*Nurse participation in hospital affairs*’).

### 4.4. Data Collection

Data were collected from December 2023 to September 2024. Researchers and trained nurse collaborators administered pencil‐and‐paper questionnaires at the selected hospitals and units, facilitating the data collection process. Participants were given 2–3 weeks to complete the questionnaires. The front page of each questionnaire included information about the purpose of the study, emphasising its voluntary and anonymous nature. Participants returned the complete questionnaires in a sealed envelope placed in the unit’s office. Before data collection began, researchers held an in‐person or telephone conversation, with each participating unit’s nurse manager to inform them about the scope of the study and its approval by the relevant authorities.

### 4.5. Ethical Considerations

The study was approved by the Research Ethics Committee of the University of Patras, Greece (registration number 13030/3.4.2022). Permission to access the hospital facilities was granted by the ethics committees of each hospital. Anonymity and the confidentiality of the departments involved were ensured by analysing the data at an overall level. The participating RNs and NAs were not rewarded and were assured that they could withdraw from the study at any time without consequences. In the front page of the questionnaire, it was explicitly stated that its completion and submission implied the participant’s informed consent.

### 4.6. Data Analysis

First, those questionnaires with incomplete MISSCARE survey and PES‐NWI items were excluded. Then, frequencies and percentages were used to describe categorical variables, while mean, standard deviation (±), median, minimum value and maximum value were used to describe continuous variables. Independent samples *t*‐test and Pearson’s and Spearman’s correlation coefficients were used to investigate bivariate relationships between demographic characteristics and the total scores of the scales. Multivariable linear regression analysis, with total score on scales as the dependent variable, was then performed to assess the independent effect of predictors. All variables that showed significant correlation with the two dimensions of MNC (regarding patients’ activities of daily living and patients’ current health status and their treatment) were included in the model. Statistical analysis was performed using the Statistical Package for Social Sciences software (IBM Corp. Released 2012. IBM SPSS Statistics for Windows, Version 21.0. Armonk, NY: IBM Corp.).

## 5. Results

### 5.1. Participants

**FIGURE 1:**
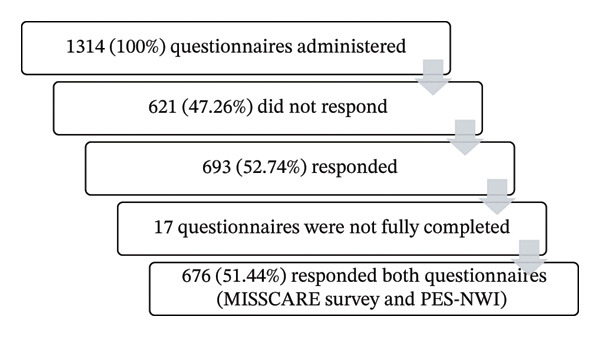
Flow diagram showing the inclusion of participants. PES‐NWI, Practice Environment Scale of the Nursing Work Index.

A total of 1314 questionnaires were distributed, and 693 were returned (response rate 52.74%). Seventeen questionnaires were excluded because they were incomplete. In total, 676 participants (51.44%) working in 28 hospitals responded to both questionnaires (Figure 1).

Most respondents (81.8%) were female, and over half were aged 35–54 years (35–44: 29.4%; 45–54: 32.8%). There were mostly RNs (60.8%), and around one‐third of them advanced educated. Overall, almost half of the participants (46.5%) had more than 10 years of professional experience. Among the participants, 62.3% worked in larger public hospitals (≥ 300 beds), and 37.7% worked in smaller hospitals (< 299 beds). Of the participants, 56.4% worked in medical units and 43.6% in surgical units (Table 1).

**TABLE 1:** Participants’ main characteristics (*n* = 676).

Variables	*Ν*	%
Gender		
Female	553	81.8
Male	85	12.6
Missing	38	5.6
Age groups		
< 25	46	6.8
25–34	130	19.2
35–44	199	29.4
45–54	222	32.8
> 54	79	11.7
Missing	0	0.0
Role		
RNs	411	60.8
NAs	265	39.2
Missing	0	0.0
RNs with Master/Doctoral education		
Yes	156	23.1
No	255	37.7
Missing	265	39.2
Employment status		
Permanent	437	64.6
Non‐permanent	224	33.1
Missing	15	2.2
Working shifts		
Morning	159	23.5
Afternoon	17	2.5
Night shift	4	0.6
Rotating shift between morning and afternoon shifts only	52	7.7
Rotating shift between morning, afternoon and night shifts	444	65.7
Missing	0	0.0
Experience in the role		
Up to 6 months	51	7.5
From 6 months to 2 years	82	12.1
From 2 years to 5 years	132	19.5
From 5 years to 10 years	97	14.3
More than 10 years	314	46.5
Missing	0	0.0
Working unit		
Medical	381	56.4
Surgical	295	43.6
Missing	0	0.0
Hospitals		
Large, ≥ 300 beds	421	62.3
Small, < 299 beds	255	37.7
Missing	0	0.0

Abbreviations: NAs, nursing assistants; RNs, registered nurses.

### 5.2. Prevalence of MNC and Reasons

As indicated in Table 2, the mean total score of the MISSCARE Survey Part A was 2.06 ± 0.65, higher in the dimensions related to patients’ daily activities (2.32 ± 0.73) than those related to the health and treatment (1.80 ± 0.63).

**TABLE 2:** MISSCARE Survey Part A: scores of dimensions and overall score (*n* = 676).

Dimensions	Mean^‡^	SD	Median	Min/max
Missed Nursing Care Related to Patients’ Activities of Daily Living	2.32	0.73	2.33	1–4.67
Missed Nursing Care Related to Patients’ Current Health Status and Treatment	1.80	0.63	1.67	1–4.83
MISSCARE Survey Part A: Total score	2.06	0.65	2.00	1–4.46

*Note*: Min = minimum, Max = maximum.

Abbreviation: SD, standard deviation.

^‡^Likert scale 1 = never missed, 5 = always missed.

The top five‐ranked missed care elements were ‘Turning patients every 2 hours’ (3.48 ± 1.15), ‘Ambulation three times per day or as ordered’ (2.89 ± 1.14), ‘Patient bathing/skin care’ (2.62 ± 1.14), ‘Mouth care’ (2.62 ± 1.13) and ‘Emotional support to patient and/or family’ (2.57 ± 1.19). The five least frequent were ‘Bedside glucose monitoring as ordered’ (1.35 ± 0.72), ‘IV/central line site care and assessments according to hospital policy’ (1.55 ± 0.78), ‘Vital signs assessed as ordered’ (1.55 ± 0.83), ‘Patient assessments performed during each shift’ (1.57 ± 0.84) and ‘Monitoring fluid intake and excretion’ (1.57 ± 0.82) (Table 3).

**TABLE 3:** MISSCARE Survey Part A: descriptive findings at the item level (*n* = 676).

Items	Mean^‡^	SD	Median	Range
Ambulation three times per day or as ordered	2.89	1.14	3	1–5
Turning patients every 2 h	3.48	1.15	4	1–5
Feeding patient when the food is still warm	2.44	1.12	2	1–5
Setting up meals for patients who feed themselves	1.93	1.07	2	1–5
Medication administration within 30 min of schedule time	2.00	1.07	2	1–5
Vital signs assessed as ordered	1.55	0.83	1	1–5
Monitoring of fluid intake and output chart	1.57	0.82	1	1–5
Full documentation of all necessary data	1.77	0.92	2	1–5
Patient teaching about illness, tests and diagnosis	2.42	1.24	2	1–5
Emotional support to patients and/or family	2.57	1.19	3	1–5
Patient personal hygiene (bathing)	2.62	1.14	3	1–5
Mouth care	2.62	1.13	3	1–5
Hand hygiene	1.72	1.01	1	1–5
Patient discharge planning	1.98	1.18	2	1–5
Bedside glucose monitoring as ordered	1.35	0.72	1	1–5
Patient assessments performed each shift	1.57	0.84	1	1–5
Focused reassessments according to patients’ conditions	1.82	0.91	2	1–5
IV/central line site care according to hospitals’ politics	1.55	0.78	1	1–5
Response to call light is initiated within 5 min	1.90	1.02	2	1–5
PRN medication requests acted on within 15 min	1.91	0.95	2	1–5
Assess effectiveness of medications	1.95	1.09	2	1–5
Assist with toileting needs within 5 min of request	2.11	1.07	2	1–5
Skin/wound care	1.66	0.84	1	1–5
Attend interdisciplinary care conference whenever held	2.13	1.35	2	1–5

*Note*: IV, intravenous.

Abbreviations: PRN, pro re nata; SD, standard deviation.

^‡^Likert scale 1 = never missed, 5 = always missed.

The overall score for reasons identified with the MISSCARE Survey Part B was 2.83 (±0.50), with average scores 3.32 (±0.56) for ‘*Staffing resources’*, 2.59 (±0.52) for ‘*Communication and teamwork*’ and 2.58 (±0.77) for ‘*Material resources*’ (Table 4).

**TABLE 4:** MISSCARE Survey Part B: scores of dimensions and total scores (*n* = 676).

Dimensions	Mean^‡^	SD	Median	Min/max
Communication and teamwork	2.59	0.52	2.64	1–4
Labor resources	3.32	0.56	3.25	1–4
Material resources	2.58	0.77	2.67	1–4
MISSCARE Survey Part B: Total score	2.83	0.50	2.85	1–4

*Note*: Min, minimum; Max, maximum.

Abbreviation: SD, standard deviation.

^‡^Likert scale 1 = not significant reason, 4 = significant reason.

According to nurses’ perceptions, ‘Inadequate number of staff’ (3.56 ± 0.67), ‘Inadequate number of assistive and/or clerical personnel’ (3.38 ± 0.71) and ‘Unexpected rise in patient volume and/or acuity on the unit’ (3.28 ± 0.73) were the most frequent reasons for MNC (Table 5).

**TABLE 5:** MISSCARE Survey Part B: descriptive findings at the item level (*n* = 676).

Items	Mean^‡^	SD	Median	Range
Inadequate number of staff	3.56	0.67	4	1–4
Urgent patients’ situation (e.g., patient’s condition worsening)	3.06	0.80	3	1–4
Unexpected rise in patient volume and/or acuity on the unit	3.28	0.73	3	1–4
Inadequate number of assistive and/or clerical personnel	3.38	0.71	3	1–4
Unbalanced patient assignments	3.05	0.82	3	1–4
Medications were not available when needed	2.47	0.91	2	1–4
Inadequate hand‐off from previous shift or sending unit	2.11	0.80	2	1–4
Other departments do not provide the care needed	2.41	0.82	2	1–4
Supplies/equipment not available when needed	2.60	0.89	3	1–4
Supplies/equipment not functioning properly when needed	2.66	0.88	3	1–4
Lack of backup support from team members	2.38	0.81	2	1–4
Tension or communication breakdowns with ancillary departments	2.54	0.79	3	1–4
Tension or communication breakdowns within nursing team	2.33	0.80	2	1–4
Tension or communication breakdown with the medical staff	2.54	0.80	3	1–4
Nursing staff did not communicate that care was not provided	2.28	0.82	2	1–4
Caregiver off unit or unavailable	2.96	0.87	3	1–4
Heavy admission and discharge activity	3.24	0.76	3	1–4

Abbreviation: SD, standard deviation.

^‡^Likert scale 1 = not significant reason, 4 = significant reason.

### 5.3. Nurses’ Perceptions of Hospital Work Environments

Overall, the total PES‐NWI score (2.53 ± 0.49) reflected a moderately supportive work environment, with strengths in managerial leadership and interprofessional collaboration but marked weaknesses in staffing adequacy (Table 6). The lowest mean score among the PES‐NWI subscales was reported for ‘*Staffing and Resource Adequacy’* (2.17 ± 0.62), indicating nurses’ perceptions of significant challenges in relation to available human and material resources. Slightly higher scores were observed for ‘*Nurse Participation in hospital Affairs’* (2.53 ± 0.58) and ‘*Nursing Foundations for Quality of Care’* (2.59 ± 0.52). ‘*Collegial nurse-physician relationship’* also received a relatively positive evaluation (2.61 ± 0.62). The highest mean was recorded in ‘*Nurse manager ability’* (2.77 ± 0.60).

**TABLE 6:** PES‐NWI scale: scores at the subscale level and total score (*n* = 676).

Subscales	Mean^‡^	SD	Median	Range
Nurse participation in hospital affairs	2.53	0.58	2.50	1–4
Nursing foundations for quality of care	2.59	0.52	2.56	1–4
Nurse manager ability	2.77	0.60	2.75	1–4
Staffing and resource adequacy	2.17	0.62	2	1–4
Collegial nurse–physician relationships	2.61	0.62	2.71	1–4
PES‐NWI: Total score	2.53	0.49	2.53	1–4

Abbreviations: PES‐NWI, Practice Environment Scale of the Nursing Work Index; SD, standard deviation.

^‡^Likert scale: 1, totally agree; 4, totally disagree.

### 5.4. Predictors of MNC

As reported in Table 7, men, permanent staff, nurses working in large public hospitals and nurses who reported worse communication and teamwork in their unit and perceived more MNC regarding patients’ activities of daily living were more likely to report MNC. Additionally, those who considered the work environment worse in terms of ‘*Nursing standards for the quality of health care*’ and ‘*Staffing and resource adequacy*’ were more likely to report MNC regarding patients’ activities of daily living.

**TABLE 7:** Multiple linear regression analysis with the dependent variable being the score on the ‘missed nursing care activities of daily living’ dimension.

Independent variables	Standardised *b* coefficient	95% CL for *b*	*p* value
Males vs. females	0.19	0.04–0.35	0.039
Non‐permanent vs. permanent staff	−0.13	−0.24–−0.02	0.019
Large hospitals vs. small hospitals	0.26	0.14–0.38	< 0.001
MISSCARE Survey Part B: Communication and teamwork^‡^	0.32	0.22–0.43	< 0.001
PES‐NWI: Nursing care standards for quality of healthcare^‡‡^	−0.31	−0.45–−0.18	< 0.001
PES‐NWI: Staffing and adequacy of resources^‡‡^	−0.21	−0.33–−0.10	< 0.001

*Note*: *b*, beta; *p*, *p*‐value.

Abbreviations: CL, confidence limits; PES‐NWI, Practice Environment Scale of the Nursing Work Index.

^‡^Likert scale: 1, not significant reason; 4, significant reason.

^‡‡^Likert scale: 1, totally agree; 4, totally disagree.

As shown in Table 8, for MNC regarding patients’ current health status and treatment, the results indicated that men, nurses aged over 45 years, permanent nurses, those working in large hospitals and those who reported worse communication and teamwork in their unit or fewer material resources also reported more MNCs. Furthermore, nurses who considered the work environment in their unit worse in terms of ‘*Nursing standards for the quality of health*’ reported more MNCs.

**TABLE 8:** Multiple linear regression analysis with the dependent variable being the score on the ‘missed nursing care patients’ current health status and their treatment’ dimension.

Independent variables	Standardised *b* coefficient	95% CL for *b*	*p* value
Males vs. females	0.19	0.05–0.31	0.007
Age ≥ 45 vs. < 45	−0.12	−0.21–−0.02	0.018
Non‐permanent vs. permanent staff	−0.18	−0.28–−0.07	0.001
Large vs. small hospitals	0.23	0.13–0.33	< 0.001
MISSCARE Survey Part B: Communication and teamwork^‡^	0.20	0.09–0.31	< 0.001
MNC Part B: Material resources^‡^	0.08	0.01–0.16	0.033
PES‐NWI Nursing care standards for quality of healthcare^‡‡^	−0.31	−0.45–−0.18	< 0.001

*Note*: *b*, beta; *p*, *p*‐value.

Abbreviations: CL, confidence limits; PES‐NWI, Practice Environment Scale of the Nursing Work Index.

^‡^Likert scale: 1, not significant reason; 4, significant reason.

^‡‡^Likert scale: 1, totally agree; 4, totally disagree.

## 6. Discussion

The findings of this national study capture nursing personnel’s perceptions of the phenomenon of MNC in Greece and examine its association with the working environment of RNs and NAs in medical and surgical units across hospitals at all levels within the National Health System, covering six out of seven health districts. In addition to MNC, the study used the PES‐NWI scale to assess the quality of the nursing work environment, providing a more comprehensive understanding of the factors influencing care delivery.

### 6.1. MNC Prevalence and the Quality of the Work Environment

The results revealed that the prevalence of MNC in Greek hospitals (overall mean 2.06) is not particularly high compared with that of international literature, both before [19] and after the pandemic [21]. However, MNC was more prevalent in relation to patients’ daily activities (mean = 2.32) and less so regarding health status and treatments (mean = 1.80), suggesting that nurses tend to prioritise clinical activities over the fundamentals of care, as also reported internationally [19].

For the PES‐NWI, the overall mean total score is 2.53, reflecting a tendency to ‘agree’ with the tool’s statements and suggesting a favourable hospital environment. The notably low score for *‘Staffing and Resource Adequacy’* is consistent with international findings, indicating that this subscale is persistently the most challenged component of the PES‐NWI. A recent meta‐analysis of 160 studies across 38 countries confirmed *‘Staffing and Resource Adequacy’* as the weakest domain internationally [22]. In contrast, other subscales, such as ‘*Nursing Foundation for Quality of Care’*, ‘*Nurse-physician Collegial Relations*’ and ‘*Nurse manager ability*’, generally achieve higher means in the range of 2.76–2.84 [23]. This pattern is also evident in Greece, where almost all subscales were rated unfavourably, particularly ‘*Staffing and Resource Adequacy*’, when compared with Magnet and non‐Magnet hospitals in the United States [24]. Similarly, Japanese studies have documented comparably low scores for this domain (mean = 2.13), highlighting persistent concerns regarding staffing adequacy across diverse health systems [25]. Collectively, these findings underscore the urgent need for systemic interventions to improve nurse staffing and resource allocation worldwide.

### 6.2. MNC Predictors

Research into the demographics and professional predictors of MNC reveals a complex interplay between individual nursing staff characteristics and organisational factors, which influence the frequency and nature of missed care. Our findings indicated that, compared to their female counterparts, male nurses, staff members younger than 45 years, permanent employees and those employed in large hospitals reported a greater prevalence of MNC. Gender differences have been reported in previous studies; for instance, Ausserhofer et al. found that female nurses were less likely to omit aspects of care than their male counterparts, suggesting potential differences in care prioritisation and professional commitment [26]. This finding was supported by Saqer and AbuAlRub (2018), who identified that female nurses and older practitioners reported lower levels of MNC in a Jordanian sample [27]. Overall, these findings may be interpreted in light of gendered socialisation processes and role expectations that promote greater attentiveness and responsibility in female nurses [28].

Age also appears to be a critical factor influencing MNC, though results are inconclusive. Studies have documented that younger nurses (< 45 years) tend to report higher frequencies of MNC [29, 30], often attributed to inexperience and limited clinical judgement skills, which may lead to difficulties with workload management and prioritisation under demanding conditions. Moreover, the concept of cognitive schemas and working memory capacity may offer a theoretical framework: experienced nurses have developed intuitive decision‐making processes and can swiftly assess complex clinical situations [31]. In contrast, Zarate‐Grajales et al. found that older nurses (> 50 years) had double the likelihood of missing basic care elements compared to those under 30, perhaps reflecting fatigue, burnout or entrenched work habits that resist adaptation to evolving clinical demands [32].

Employment status and staffing patterns have been strongly linked to MNC. Permanent staff nurses tend to report higher levels of missed care than temporary or auxiliary personnel [33]. Permanent staff often bear the responsibility of supervising less experienced auxiliary workers, increasing their workload and stress. Temporary staff, while less experienced clinically, might not be as engaged in direct care tasks or may avoid reporting omissions. Estabrooks et al. identified a correlation between higher proportions of temporary nursing staff and increased inpatient mortality rates in Canada [34]. This association likely reflects the lower experience and clinical judgement of temporary nurses, who are less equipped to use heuristic and intuitive processes to manage patient care safely and efficiently [35]. Increased use of temporary staff was associated with higher reported rates of MNC, emphasising the importance of stable, experienced staffing in ensuring care quality [36].

The type of healthcare facility also shapes the prevalence of MNC. Nurses working in large hospitals tend to report higher levels of missed care than those smaller facilities [37]. This difference can be attributed to the increased bed capacity, complexity of cases and diversity of specialised procedures common to tertiary hospitals, which place greater demands on nursing staff. Higher patient acuity and more complex therapeutic regimens increase the risk of care omissions, especially when coupled with staffing shortages or inadequate support [7].

Another significant factor associated with MNC is the presence or absence of clear evidence‐based nursing foundations for quality care. In this study, the ‘*Nursing Foundations for Quality Care*’ subscale of the PES‐NWI assessed perceptions of the quality of nursing standards within hospital units (e.g., availability of up‐to‐date protocols, emphasis on quality assurance and the extent to which nurses are encouraged to deliver evidence‐based care) [17]. Environments characterised by strong professional practice standards, shared governance and nursing autonomy are associated with lower rates of MNC [38]. In contrast, workplaces with vague expectations, minimal quality oversight tend to foster care fragmentation and disengagement among nursing staff. Our findings showed that poor perceptions on this subscale were significantly associated with increased MNC across both domains examined. Low scores on this subscale often indicate insufficient institutional support for quality improvement, a lack of continuing education opportunities or inadequate mechanisms for feedback and evaluation. Such environments create conditions in which nurses are more likely to feel unsupported and overwhelmed, increasing the incidence of task omissions [39]. In Greece, the implementation of nursing standards varies widely between institutions and regions, with many nurses reporting limited involvement in decision‐making and minimal emphasis on clinical quality indicators. Consequently, even when nurses are motivated to provide comprehensive care, the absence of structural standards limits their ability to do so consistently and safely [12, 39]. Addressing this gap requires a dual strategy: first, the development and dissemination of national nursing care standards aligned with international best practice; second, fostering a supportive work environment where quality care is both expected and facilitated. Institutions that invest in nurse‐led quality improvement initiatives, clinical mentoring and transparent communication channels typically report better care outcomes and reduced MNC [40]. Moreover, ensuring that nurses have access to sufficient clinical tools, clear documentation systems and protocols based on current evidence enhances their ability to deliver all aspects of patient care. When combined with adequate staffing, strong nursing care standards form the foundation for safe, effective and patient‐centred healthcare.

### 6.3. Inadequate Staffing as the Main Predictor for MNC

Our findings indicate that inadequate staffing is the most prominent factor associated with MNC, significantly higher than both ‘*Communication and teamwork*’ and ‘*Material resources*’. These results are consistent with most international studies [41]. Adequate nurse staffing has consistently been identified as a key determinant in reducing MNC [42]. Systematic reviews further corroborate the central role of labour resources, particularly insufficient staffing, workload pressure and sudden patient surges, as consistent predictors of MNC across diverse health systems [43, 44]. This indicates that, in situations where resources are insufficient to provide the required care to all patients, nurses resort to an informal prioritisation process to allocate care. Adequate staffing of nursing and other personnel is therefore a fundamental organisational characteristic of the work environment, and improved hospital staffing significantly reduces both the frequency and consequences of MNC [44].

Although better staffing indirectly enhances communication and teamwork among healthcare professionals, current evidence suggests that these relational aspects, while important for overall care quality, are not as strong predictors of MNC as staffing adequacy itself. Deficiencies in teamwork and poor interprofessional collaboration contribute to errors, delays and fragmented care, yet the association with MNC appears to be secondary compared to the effect of inadequate nurse‐to‐patient ratios [45, 46]. On the other hand, effective teamwork and communication can mitigate the negative impact of low staffing levels by improving coordination and ensuring that essential care tasks are less likely to be omitted [47]. For instance, Kalisch et al. emphasised that stronger teamwork among nursing staff was associated with lower reports of MNC, even in high workload settings, whereas Griffiths et al. argued that structural constraints such as staffing shortages outweighed the benefits of improved communication [3, 4]. Our findings support the interpretation that while labour shortages are the most universal and consistent determinant of MNC, contextual variables may elevate the role of communication and material resources in specific settings.

According to the latest OECD data [9], Greece reported 2.23 RNs and NAs per 1000 population in 2024, a figure that places the country significantly below the EU27 average of 7.52 and far behind leading European countries such as Norway (16.52), Finland (14.13) and Ireland (13.30). In hospital environments with chronic understaffing, nurses are forced to engage in clinical prioritisation, a process through which tasks are informally ranked by urgency. While urgent treatment‐related procedures may be prioritised, equally important yet less urgent care aspects such as patient mobilisation, hygiene or emotional support are more likely to be left incomplete or delayed. From a policy perspective, this underinvestment in the nursing workforce contributes to increased workload, higher burnout rates and compromised quality of care. It also limits the capacity of the system to implement nurse‐led models of care and to manage chronic conditions effectively, especially in rural and underserved regions.

To align with European standards and ensure sustainable healthcare delivery, Greece must significantly increase its nursing workforce, both in absolute numbers and in strategic development. Reforms should also address structural barriers to nursing practice environments. However, improving staffing levels remains a challenge in Greece’s healthcare system due to ongoing financial limitations and national nursing shortages.

### 6.4. Limitations

This study has several limitations. The cross‐sectional design does not permit causal inferences between reasons and MNC occurrence. Additionally, the use of self‐reported questionnaires may have introduced response bias, as nurses might underreport or overreport omissions. Although the study was national, the convenience sampling method and focus on public hospitals may limit the generalisability of the findings to the broader healthcare system, including private sector facilities. Furthermore, the inclusion of only approximately a quarter of Greek hospitals may further limit the generalisability of the findings. A more inclusive approach, also adopting longitudinal designs and objective measurements, is recommended to corroborate, expand and generalise the findings.

## 7. Implications for Nursing Management

To the best of our knowledge, this study is the first to examine MNC in Greek hospitals in a consistent manner, using validated and long‐established instruments. Our findings highlight important implications for nursing management. Inadequate staffing remains a critical barrier to the consistent delivery of safe and comprehensive care, reinforcing the need for strategic workforce planning and investment in nurse recruitment and retention. At the same time, the establishment and reinforcement of foundational standards of nursing care are essential to ensure that critical practice is guided by evidence‐based protocols, clear quality benchmarks and supportive governance structures. Nursing managers must therefore prioritise both adequate staffing levels and the systematic implementation of nursing care standards, as these elements together form the cornerstone of effective, patient‐centred healthcare.

## 8. Conclusion

This study provides an overview of the MNC phenomenon and its related factors in Greece. MNC is influenced by a combination of demographic (gender, age), professional (experience, employment status) and organisational factors (type of hospital, staffing patterns). Overall, inadequate staffing appears to be the most critical predictor of MNC in Greek public hospitals.

In Greece, where staffing levels remain significantly below European and OECD averages, addressing the nursing workforce shortage is an urgent priority. Strategic investments in recruiting, retaining and appropriately deploying nursing staff are essential to reduce MNC, enhance patient care and strengthen the overall health system. However, addressing MNC requires multifaceted strategies that consider nurse demographics, experience and organisational support systems, ultimately aiming to optimise nursing care delivery and patient safety. Chronic nurse understaffing, resulting in high patient‐to‐nurse ratios, creates a persistent barrier to the delivery of comprehensive, high‐quality care. Inadequate staffing not only increases the frequency of MNC in essential daily care activities but also contributes to poor teamwork, communication breakdowns and weakened adherence to nursing care standards. These structural deficiencies may further compromise patient safety and outcomes. Policymakers must recognise that without sufficient and well‐supported nursing personnel, efforts to improve healthcare quality, patient safety and workforce sustainability will remain incomplete and ineffective.

## Author Contributions

Alexandra Giannarou: formal analysis, investigation, data curation, writing–original draft, writing–review and editing, and visualisation.

Michael Igoumenidis: conceptualisation, methodology, validation, formal analysis, data curation, writing–review and editing, and supervision.

Nikos Stefanopoulos: conceptualisation, methodology, validation, formal analysis, data curation, nd writing–review and editing.

Anastasios Tzenalis: writing–review and editing.

Petros Galanis: formal analysis, writing–review and editing.

Stefania Chiappinotto: validation, data curation, and writing–review and editing.

Alvisa Palese: conceptualisation, methodology, validation, data curation, writing–review and editing, and supervision.

## Funding

No funding was received for this manuscript. Open access publishing facilitated by Universita degli Studi di Udine, as part of the Wiley ‐ CRUI‐CARE agreement.

## Conflicts of Interest

The authors declare no conflicts of interest.

## Data Availability

The data that support the findings of this study are available from the corresponding author upon reasonable request.
